# Dr. Waldo Elias Boardman

**Published:** 1922-10

**Authors:** 


### OBITUARY

### DR. WALDO ELIAS BOARDMAN

Dr. Waldo Elias Boardman died August 14, 1922, in Omaha, Nebraska. Dr. Boardman was 70 years of age when he died.

Dr. Boardman and his wife had been attending the meeting of the American Dental Association in Los Angeles, and became ill in Omaha, where he died in the Methodist Hospital of Omaha. Dr. Boardman studied dentistry, after having engaged in several lines of commercial business, and graduated from the Dental Department of Harvard University, June 29, 1886. He was at one time an instructor in operative dentistry for about ten years. He developed a successful private practice, but always retained his interest in the college work. At his death he was a valued member of the administrative board of Harvard. He gave much time and effort to the collection of a dental library, which at the present time is one of the best college libraries in this country.

Dr. Boardman was devoted to dentistry, and gave much time and effort to the cultivation of our professional affairs. He was a constant attendant of the American Dental Society, as he was of all local societies of the vicinity in which he resided. Few men have attended so many dental society meetings, and none have been more devoted to the best interests of the profession.

He was of sterling character and an ideal professional gentleman. He stood always for the highest ideals and had no sympathy with popular and mercenary activities. He wrote many papers on dental topics and was always much interested in papers and their discussions, in which he participated generously, and always thoughtfully and learnedly.

He took much interest in the civic affairs of Boston and always did his part. He was highly esteemed by all who knew him. The dental profession has lost one of its strong and worthy devotees, and we shall miss his wise and helpful ministrations. We shall long cherish his gracious memory, and with much profit to our own higher aspirations for our devoted profession.

